# Use of filter disks or sinus packs for single and repeated measurement of total IgE and house dust mite specific IgE in nasal secretions

**DOI:** 10.1186/2045-7022-5-S4-P18

**Published:** 2015-06-26

**Authors:** Margot Berings, Natalie De Ruyck, Gabriële Holtappels, Claus Bachert, Philippe Gevaert

**Affiliations:** 1 Ghent University, Upper Airway Research Laboratory, Ghent, Belgium

## Background

There is evidence for local production of IgE in the nasal mucosa of patients with allergic rhinitis (AR). Collection of nasal secretions (NS) by validated methods for measurement of local IgE is of great interest. We aimed to compare two methods for collection of NS - sinus packs (SP) and filter disks (FD) - and to evaluate the suitability of using a fixed dilution instead of a fixed volume when processing.

## Method

In a first experiment, NS were collected by means of FD and SP in 15 HDM AR patients and 15 controls. During processing, saline solution was added in order to mobilize the NS. For each sample the volume to be added was calculated in order to obtain a fixed dilution. In a second experiment, NS from 2 HDM AR patients were collected by means of FD and SP on 4 consecutive days. This experiment was performed once with fixed volume and once with fixed dilution. At last, repeated collection of NS was performed by means of FD and SP in 5 HDM AR patients on several time points before and during HDM SCIT. Immunoglobulin levels were measured with the UniCap system.

## Results

In the first experiment, levels of total IgE and HDM sIgE were significantly higher in HDM AR patients compared to controls. With the FD, total IgE and HDM sIgE were below the detection limit (BDL) in 2 and resp. 6 out of 15 AR patients. With the SP, total IgE and HDM sIgE were BDL in 3 and resp. 4 out of 15 AR patients. When comparing FD and SP in the same patients on 4 consecutive days, more variation in IgE levels was seen with the FD. When comparing a fixed volume to a fixed dilution, the latter is more time consuming and prone to error. The fixed dilution brings along a risk of excessive dilution when larger volumes are added, and insufficient mobilization of NS when smaller volumes are added. In the third experiment, an increase in HDM sIgG and sIgG4 was observed in serum of 4 resp. 3 out of 5 patients during HDM SCIT. In 3 resp. 2 out of 5 patients, an increase in HDM sIgG and sIgG4 was also observed in their NS collected by means of SP.

## Conclusion

Overall, SP seem to be superior to FD in many aspects. However, SP cause stimulation of the nasal mucosa, making them not suitable for serial measurements on the same day. Fixed dilution raised several difficulties and was not superior to a fixed volume. Using SP with addition of a fixed volume of saline seems to be the most reproducible method to measure total and allergen specific IgE, IgG, and IgG4 in clinical trials.

